# Myocardial T1 responds to adenosine - normal values of stress T1 reactivity at 1.5T and 3T

**DOI:** 10.1186/1532-429X-17-S1-P107

**Published:** 2015-02-03

**Authors:** Alexander Liu, Rohan S Wijesurendra, Jane M Francis, Matthew D Robson, Stefan Neubauer, Stefan K Piechnik, Vanessa M Ferreira

**Affiliations:** Oxford Centre for Magnetic Resonance Research (OCMR), University of Oxford, Oxford, UK; Functional MRI of the Brain (FMRIB) centre, University of Oxford, Oxford, UK

## Background

Myocardial vasodilator reserve is an important surrogate marker of normal and abnormal cardiac physiology. Pharmacological vasodilator agents such as adenosine increase myocardial blood volume, which is expected to prolong the observed T1-relaxation. We explored the novel application of stress T1-mapping by assessing the response of myocardial T1 to the administration of adenosine in healthy volunteers.

## Methods

Healthy volunteers without history of cardiovascular disease, not on cardiovascular medications with a normal ECG underwent CMR studies at 1.5T (n=10, 33±10 years) and 3T (n=10, 36±11 years). T1-maps were acquired using the shortened Modified Look-Locker Inversion recovery (ShMOLLI, Piechnik, JCMR 2010, 12:69) sequence at rest and under adenosine stress (140 μg/kg/min IV for at least 3 min) in 3 short-axis (basal, mid-ventricular, apical) slices. Mean T1 values were derived for whole-heart, per-slice and segmental analyses (AHA 16-segment model). Stress/rest first-pass myocardial perfusion imaging was performed in matching short-axis slices. Left ventricular function and viability were assessed by CINE and late gadolinium enhancement (LGE), respectively.

## Results

**Figure 1:**
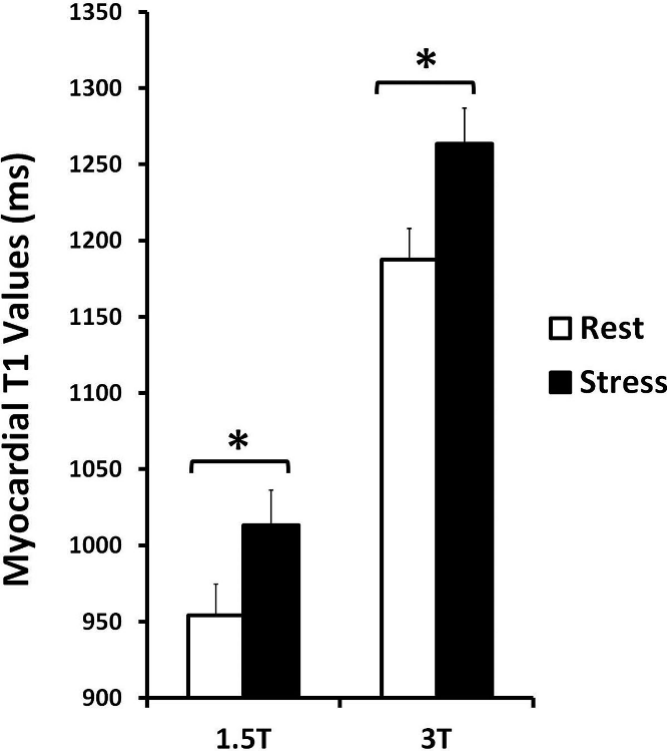
**In healthy volunteers, myocardial T1 values at rest increase significantly under adenosine stress at both 1.5T and 3T.** Error bars represent 1 standard deviation from the mean T1 values. * denotes p<0.0001.

**Figure 2:**
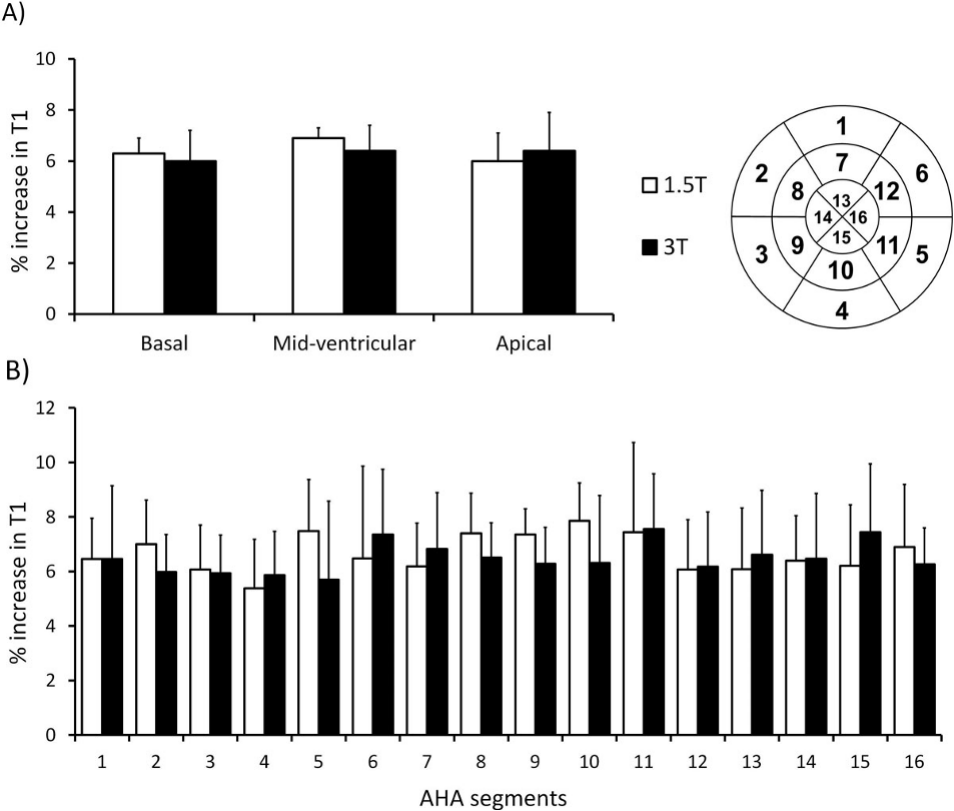
**There is no significant variation in the myocardial T1 response to adenosine stress on a per-slice (A) or according to the American Heart Association (AHA) 16-segment model (B).** Error bars represent 1 standard deviation from the mean T1 values. All ANOVA p >0.16.

All healthy volunteers had normal left ventricular function (66±5%), myocardial perfusion reserve indices (2.0±0.3) and no LGE. Mean resting ShMOLLI T1 values were normal: 954±20 ms (1.5T) and 1188±33 ms (3T). Compared to rest, mean myocardial T1 values under adenosine stress increased significantly at both 1.5T and 3T (both p<0.0001, figure 1), with no significant dependencies on field strength (6.2 ± 2.0% vs 6.4 ± 1.9%, respectively, p=0.60) or variation between slices or AHA segments (all p>0.16 by ANOVA, Figure 2).

## Conclusions

Myocardial T1 increases significantly in response to adenosine vasodilator stress in normal controls. The relative T1 change of ~6% is independent of conventional field strengths (1.5/3T) and myocardial slice or segment positioning. This is likely due to the inherent intra-scan compensation of reported known variation sources in T1-mapping. With the presumed vascular origin of the response, the stress T1 holds promise for becoming a novel biomarker for mapping regional ischaemia and for detecting significant coronary artery disease in future studies.

## Funding

The research was supported by the National Institute for Health Research (NIHR) Oxford Biomedical Research Centre based at the Oxford University Hospitals NHS Trust, University of Oxford. AL is a National Institute for Healthy Research (NIHR) funded Academic Clinical Fellow, UK. SN acknowledges support from the British Heart Foundation Centre of Research Excellence, Oxford.

